# Antibiotic Treatment Does Not Ameliorate the Metabolic Changes in Rats Presenting Dysbiosis After Consuming a High Fructose Diet

**DOI:** 10.3390/nu12010203

**Published:** 2020-01-13

**Authors:** Ariel Bier, Rawan Khasbab, Yael Haberman, Tzipi Braun, Rotem Hadar, Katya Sosnovski, Amnon Amir, Avshalom Leibowitz, Ehud Grossman

**Affiliations:** 1 Internal Medicine D and Hypertension Unit, The Chaim Sheba Medical Center, Tel-Hashomer, Ramat Gan 5265601, Israel; arielbier@gmail.com (A.B.); rawankhasbab@mail.tau.ac.il (R.K.); Ehud.Grossman@sheba.health.gov.il (E.G.); 2 Sackler Faculty of Medicine, Tel-Aviv University, Tel Aviv 69978, Israel; Yael.Haberman@sheba.health.gov.il; 3 The Chaim Sheba Medical Center, Tel-Hashomer, Ramat-Gan 5265601, Israel; zipik0@gmail.com (T.B.); rotemhadar@gmail.com (R.H.); 1katya3@gmail.com (K.S.); amnonim@gmail.com (A.A.); 4 Cincinnati Children’s Hospital Medical Center, University of Cincinnati College of Medicine, Cincinnati, OH 45229, USA

**Keywords:** fructose, metabolic syndrome, microbiome, dysbiosis

## Abstract

High fructose consumption is one of the hallmarks of Western diets and has been found to induce MeS symptoms in parallel to gut microbial dysbiosis. However, the causality between those two is still elusive. Here, we studied whether a significant modification of gut microbial composition by antibiotics can influence the fructose-induced metabolic changes. Male Sprague-Dawley (SD) rats were divided into four groups including controls, controls + antibiotics, high fructose diet (HFrD, 60% fructose), HFrD + antibiotics (n = 7–8 in each group) for a period of 8-weeks. The high fructose diet increased blood pressure (BP), triglyceride (TG), fatty liver and the expression of hepatic genes related to lipogenesis, and fructose transport and metabolism. In addition, fructose changed the microbial composition and increased acetic and butyric acids in fecal samples but not in the blood. Antibiotic treatment significantly reduced microbial diversity and modified the microbial composition in the samples. However, minimal or no effect was seen in the metabolic phenotypes. In conclusion, high fructose consumption (60%) induced metabolic changes and dysbiosis in rats. However, antibiotic treatment did not reverse the metabolic phenotype. Therefore, the metabolic changes are probably independent of a specific microbiome profile.

## 1. Introduction

Fructose consumption has significantly increased in Western diets over the last few decades, as many prepared beverages and foods have added sucrose or high fructose corn syrup [1]. Western diet consumption is linked to metabolic syndrome (MeS) and diabetes mellitus type 2 (T2DM) development, as well as their complications, including non-alcoholic fatty liver (NAFL) and diabetic nephropathy [2,3,4]. In animal models, high fructose diets rapidly cause features of MeS including: dyslipidemia, fatty liver, hypertension, insulin resistance and glucose intolerance. Short term fructose feeding in humans increases triglyceride levels and insulin resistance that are not observed with equivalent amounts of glucose [5,6,7]. The conclusion of these studies is that fructose ingestion can significantly contribute to metabolic syndrome development [8].

Ingredients in the diet can have both direct and indirect effects, and the indirect effects are thought to be mediated by the gut microbiome. The digested food influences the community, structure, and function of the gut bacteria which can result in beneficial or detrimental consequences on the host’s health. Previous studies have shown that different dietary regimens alter the microbial composition or dysbiosis in the gut, and can alter the development and maintenance of MeS [9]. Moreover, fructose consumption is specifically linked to gut microbial dysbiosis [10,11,12], suggesting that the metabolic changes are mediated by the microbiome [10,13,14].

Previous studies have used rats that were fed a 30% fructose diet with and without antibiotics [12,15,16] or underwent fecal transplantation [12] treatments. In those studies, antibiotic treatments and fecal transplantations ameliorated the fructose associated metabolic changes. These studies have shown that upon modification of the microbial composition, there was a systemic and tissue specific reduction in insulin resistance [12,16], oxidative stress [12,15], blood lipids levels [12], liver damage [15,16], and in inflammatory markers [12,15]. In addition, a study on mice consuming a 30% fructose diet has described that antibiotic treatment suppressed the hippocampal neuroinflammatory response [17].

We used a well-established metabolic syndrome rat model [18,19,20,21] where male SD rats are fed a 60% fructose containing diet, and develop MeS within several weeks, mainly characterized by elevated TG, fatty liver, and hypertension. Our aim in the present study was to test if a high fructose diet affects the gut microbial composition, and test if antibiotics that significantly reduce the gut microbial composition and diversity modify the fructose-induced metabolic changes.

## 2. Materials and Methods

### 2.1. Animals

Male SD rats, weighing 200 ± 20 g, were purchased from Envigo Laboratories, Jerusalem, Israel. The rats were housed in regular cages (two rats per cage), and were situated in an animal room at 22 °C using a 14-h light (6:00–20:00)/10-h dark (20:00–6:00) cycle with free access to food and drink. All procedures performed were in accordance with the Chaim Sheba Medical Center’s Guidelines for Animal Studies and approved by the Institutional Animal Ethics Committee (ethical approval code 1106/17). The rats were maintained on a normal chow diet and were given tap water to drink ad libitum for an acclimation period of seven days. The experimental diets were the following: the high fructose diet (HFrD) (TD.89247 Teklad Envigo Madison, WI, USA) consisted of 60% fructose. The control diet (TD.170308; Teklad Envigo Madison, WI, USA) was a custom diet matching the HFrD. A detailed comparison of the diets is provided in Table 1. The rats were then divided into four groups (n = 7–8 each): control (Ctrl), control + antibiotics (Ctrl + AB), fructose (Fructose) and fructose + antibiotics (Fructose+AB). The rats were fed over an 8-week period and upon conclusion, the rats were anesthetized with 3% isoflurane (the depth of anesthesia was confirmed by rear foot squeezing), euthanized, and their livers were harvested. The livers were weighed and body weight was determined for standardization. Several portions of the liver tissue were quickly removed, snap-frozen in liquid N_2_, and stored at −80 °C. Frozen tissues were used for RNA extraction.

### 2.2. Antibiotic Treatment

Antibiotic treatment was given in the drinking water and contained ampicillin (PanReac AppliChem A0839, 0025) 1 g/L + neomycin (Biological Industries 1405-10-3) 0.5 g/L. Ampicillin and neomycin were chosen because ampicillin is active against Gram-positive and some Gram-negative bacteria, while neomycin has excellent activity against Gram-negative bacteria and has partial activity against Gram-positive bacteria. In addition, both antibiotics are poorly absorbed (or unabsorbed as in the case of neomycin) and thus have no systemic effects [22].

### 2.3. Metabolic Parameters

Rats were weighed at baseline and every two weeks thereafter. Metabolic parameters and BP were measured at baseline and at the end of the study. Fasting blood glucose, insulin, and TG levels were measured as follows: glucose and TG levels were assayed with an automated analyzer for an enzymatic colorimetric reaction (Olympus AU 2700, Hamburg, Germany). An insulin ELISA kit (10-1250-01, Mercodia AB, Uppsala, Sweden) was used to measure insulin levels. The HOMA-2 IR index was calculated by a free online calculator (HOMA Calculator, Version 2.2.3, Diabetes Trial Unit, Oxford University, Oxford, UK). Systolic BP was measured by the indirect tail cuff method, using an electrosphygmomanometer and a pneumatic pulse transducer (58,500 BP Recorder, UGO BASILE, Varese, Italy). The mean of five consecutive readings determined systolic BP.

### 2.4. Liver Studies

#### 2.4.1. Liver Histological Studies

Liver tissue slices were fixed in 4% paraformaldehyde and embedded in paraffin for histological studies. Sections from the paraffin embedded liver were stained with hematoxylin and eosin for steatosis assessment. A validated scoring system was used for the quantification of fatty liver changes [23]. Evaluations were performed by a pathologist blinded to the treatment groups. Images were captured by an Olympus BX50 microscope equipped with a digital camera (Olympus DP71, Olympus Europa SE & CO. KG, Hamburg, Germany).

#### 2.4.2. Real-Time Quantitative Reverse Transcription PCR of Liver Genes

mRNA expression levels of genes were ascertained in the liver tissue by real-time quantitative reverse transcriptase –PCR (qRT-PCR). Total RNA was extracted from the liver tissue using a NucleoSpin RNA Kit (Macherey-Nagel, Düren, Germany). Reverse transcription was performed using an Applied Biosystems High Capacity cDNA Reverse Transcription Kit (Applied Biosystems, Foster City, CA, USA). qRT-PCR reactions were performed using the Power SYBR Green PCR Master Mix (Applied Biosystems, Warrington, UK) and the Applied Biosystems 7500 real-time PCR system. The cycling condition were: first single step of 95 °C for 0.20 min and then 40 cycles of melt stage of 3 s for 95 °C and a extend stage of 30 s for 60 °C. At the end of the 40 cycles, a melt curve stage was done. The ribosomal protein lateral stalk subunit P0 (Rplp0) mRNA was used as an internal control. The primers are listed in Table 2.

### 2.5. SCFA Extraction and Analysis

Flash-frozen fecal contents (100 mg) were mashed in 2 mL acidic water (pH = 2.4) and centrifuged at 12,100× *g* for 20 min at 4 °C, after which the supernatants were taken for analysis. The samples were mixed with 10 µmoL/g internal standards (acetic-d4 acid, Sigma-Aldrich, St. Louis, MO, USA, #151785; propionic-3,3,3-d3 acid, #486159; and butyric-d8 acid, #588555). Two rounds of extraction using 1mL hexane were carried out by mixing for 10 min at room temperature, followed by centrifugation at 1932× *g* for 10 min at 4 °C. Extracts were then incubated at 65 °C for 45 min with N-tert-Butyldimethylsilyl-N-methyltrifluoroacetamide (MTBSTFA) (Sigma-Aldrich), 6 µL in 180 µL sample. For blood samples, 50 µL plasma was diluted with 300 µL PBS + 35 ul HCl. The samples were mixed with 1.25 µmol/50 µL internal standards (acetic-d4 acid, Sigma-Aldrich #151785; propionic-3,3,3-d3 acid, #486159; and butyric-d8 acid, #588555). Two rounds of extraction using 750 µL ether were carried out by mixing for 1min at room temperature, followed by centrifugation at 12,100× g for 3 min at 40 °C. Extracts were then incubated at 65 °C for 2 h with N-tert-butyldimethylsilyl-N- methyltrifluoroacetamide (MTBSTFA) (Sigma-Aldrich) 9 µL in 180 µL sample. Gas chromatography-mass spectrometry (GC-MS) analyses were performed using an Agilent 6890/5977A GC-MS (Santa Clara, CA, USA) system equipped with Agilent 30 m × 0.25 mm i.d. HP-5MS UI column (5% Phenyl/Methylpolysiloxane, 0.25 µm film thickness). The carrier gas was helium (99.999%) at a constant flow rate of 1.0 mL/min. The GC conditions were as follows: injection volume 1.0 µL (Agilent auto-sampler G4513A, Beijing, China); injector temperature 250·°C in split less mode; the initial oven temperature was 60 °C, which was maintained for 4 min, and increased to 80 °C at a rate of 2 °C/min, which was followed by raising the temperature to 260 °C at a rate of 30·°C/min with holds for 3 min. MS was performed in the EI positive ion mode at 70 eV electron energy. Transfer line temperature and ion source temperature were maintained at 280 °C and 250 °C, respectively. MS data were collected in full-scan mode (m / z 60-300) and analyzed with Agilent Chemstation software (Agilent Technologies, Ver. F.01.01.2317). The m/z of reconstructed single ion monitoring (RSIM) are as follows: 117 (acetic acid), 120 (acetic-d4 acid), 131 (propionic acid), 134 (propionic-3,3,3-d3 acid), 145 (butyric acid), and 152 (butyric-d7 acid).

### 2.6. Microbiome Analysis

#### 2.6.1. DNA Extraction, PCR Amplification, and Sequencing

DNA extraction and PCR amplification of the variable region 4 (V4) of the 16S rRNA gene using Illumina adapted universal primers 515F/806R39 was conducted using the direct PCR protocol [Extract-N-Amp Plant PCR kit (Sigma-Aldrich, Inc.)] as previously described [24,25,26]. Briefly, PCRs were conducted in triplicate in 96 wells plate [denaturation for 3 min at 94 °C; 35 cycles (98 °C, 60 s; 55 °C, 60 s; 72 °C, 60 s) followed by elongation for 10 min at 72 °C]. Positive amplicons were pooled in equimolar concentrations into a composite sample that was size selected (300–500 bp) using agarose gel to reduce non-specific products from host DNA. Sequencing was performed on the Illumina MiSeq platform with the addition of 20% PhiX, and generating paired-end reads of 175b in length in each direction.

#### 2.6.2. Microbiome Data Processing and Analysis

Reads were processed in a data curation pipeline implemented in QIIME 2 version 2019.4 [27]. Reads were demultiplexed according to sample specific barcodes. Quality control was performed by truncating reads after three consecutive Phred scores lower than 20. Reads with ambiguous base calls or shorter than 150 bp after quality truncation were discarded. Sequence variants (SV) detection was performed using Deblur [28]. Reads were then truncated to 150 bp. SVs taxonomic classification was performed using a naive Bayes fitted classifier, trained on the August 2013 99% identity Greengenes database, for 150 bp long reads and the Forward/Reverse primer set. Unweighted UniFrac was used as a measure of β-diversity = between sample diversity [29], using a phylogenetic tree generated by SEPP [30]. All samples were rarefied to 6000 reads for β diversity analysis, to avoid sample size affect. The resulting distance matrix was used to perform a principal coordinate analysis (PCoA). A heatmap was generated using Calour version 2018.10.1 with default parameters [31]. MaAsLin2 (Multivariate Association with Linear Models) R package version 0.99.2 [32] was used with default parameters to find SVs significantly associated with diet, with and without antibiotics. Only significant associations with q ≤ 0.05 after a false discovery rate (FDR) correction were included.

### 2.7. Statistical Analysis

Data are presented as mean ± S.E.M. Statistical analysis was performed using the IBM^®^ SPSS^®^ Statistics, version 24 (IBM Corporation, Armonk, NY, USA). One-way analysis of variance (ANOVA) and the post hoc Tukey method examined the differences between groups. Real-time qPCR data were analyzed using DataAssist software, version 3.01 (Applied Biosystems, Life Technologies Corporation 2012, now under Thermo Fisher Scientific, Waltham, MA, USA). A *p* value of ≤0.05 was considered statistically significant.

## 3. Results

### 3.1. Metabolic and Hemodynamic Effects

**Figure 1:**
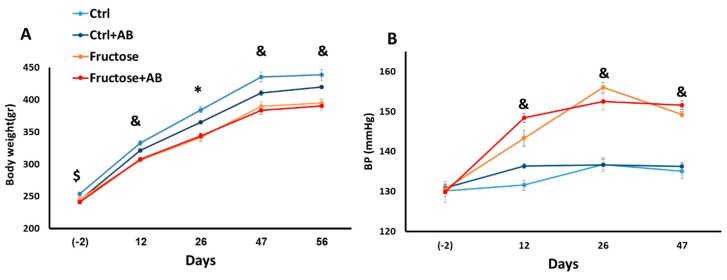
Fructose induces hypertension even with antibiotic treatment. Body weight was measured before fructose diet and on days 12, 26, 47 and 56 after diet onset (**A**). Blood pressure was measured before the fructose diet and on days 12, 26 and 47 after diet onset (**B**). *—*p* ≤ 0.05 Ctrl + AB, fructose and fructose + AB versus Ctrl. &—*p* ≤ 0.05 fructose and fructose + AB versus Ctrl and Ctrl + AB. $—*p* ≤ 0.05 fructose + AB versus Ctrl.

**Figure 2:**
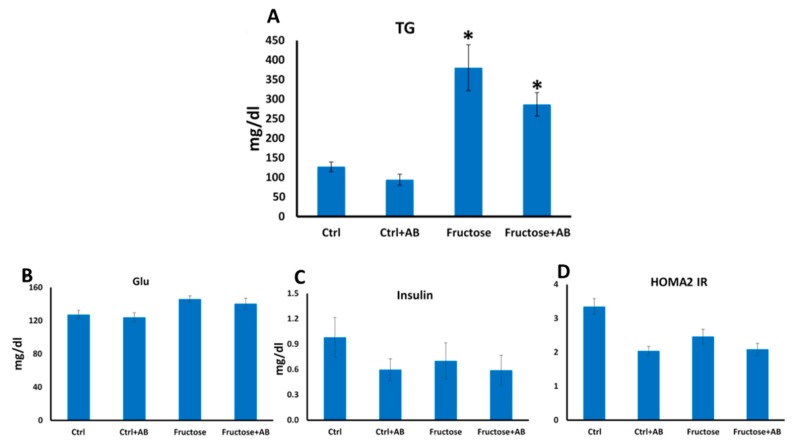
Fructose induces hypertriglyceridemia even with antibiotic treatment. Blood triglycerides were measured at the end of the experiment. (**A**). Blood glucose and insulin were measured at the end of the experiment and the HOMA IR index was calculated (**B**–**D**). *—*p* ≤ 0.05 versus Ctrl.

Similar to previous work [21], HFrD reduced bodyweight in rats. However, this observation is inconclusive, since the control group was slightly heavier than all other groups at baseline (Figure 1A). HFrD increased BP and plasma TG and antibiotics did not prevent these increases (Figure 1B and Figure 2A). HFrD had no effect on blood glucose, insulin and on the HOMA2 IR index (Figure 2B–D).

### 3.2. The Microbiome Profile

**Figure 3:**
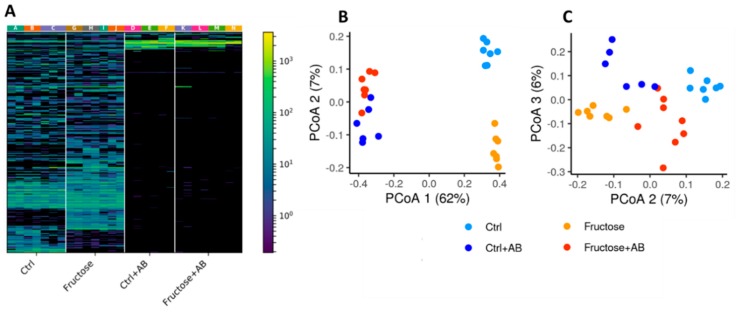
Microbial composition of high fructose diet and control rats, with and without antibiotics. (**A**) A heatmap representing all sequence variants (SV) in all samples. Each column represents a different SV and each row a different sample. Samples are clustered by group and SVs are clustered using hierarchical clustering in color. The different cages in which the rats lived are marked A-N. (**B**,**C**) Unweighted UniFrac PCoA plot representing all samples in this cohort, colored by diet and antibiotics intake. PC1-PC2 (**B**) and PC2-PC3 (**C**) are shown.

Antibiotic intake had the strongest effect on the microbiome composition, as it depleted most but not all microbial taxa (Figure 3A). The high fructose diet also substantially affected the overall microbial composition as demonstrated in the lower part of the heatmap (Figure 3A). To further visualize the similarities and variations between the samples’ microbial composition, an unweighted UniFrac based PCoA of the cohort was performed. Again, antibiotic intake had the strongest effect, with a strong difference in PC1 between samples with and without antibiotics (Figure 3B). Control and fructose samples also have strong clustering separation, mostly on PC2 (Figure 3B,C). These results do not seem to be biased by shared cages, as there were multiple cages per group and no strong cage-related batch effect can be observed (Figure 3A).

**Figure 4:**
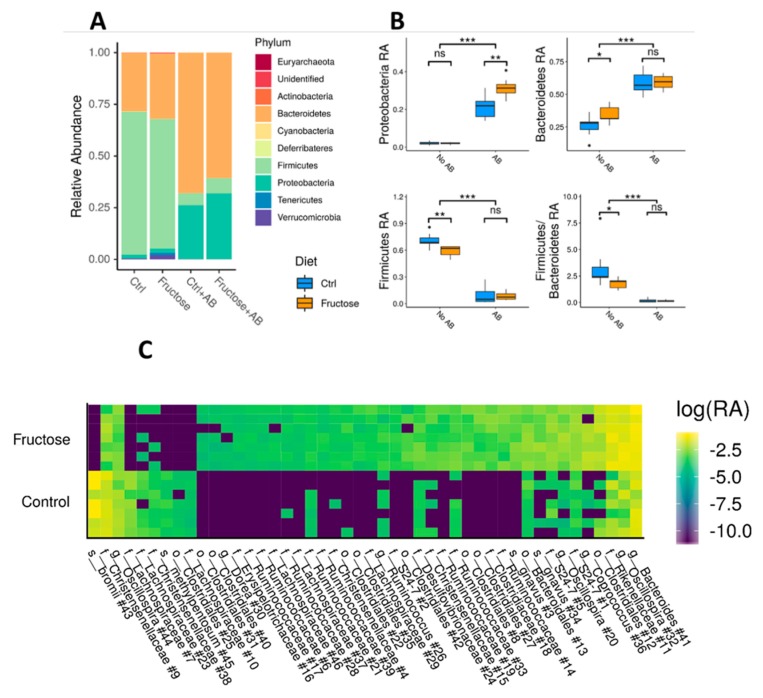
Specific taxonomic differences between groups. (**A**) Median taxonomic relative abundance at the phylum level, by diet and antibiotics intake. (**B**) Boxplots of Proteobacteria, Firmicutes, Bacteroidetes, and Firmicutes/Bacteroidetes relative abundance (RA), between rats that took antibiotics and rats that did not, both for high fructose diet and controls. Asterisks indicate significant differences (Mann Whitney test: ***—*p* < 0.001, **—*p* < 0.01, *—*p* < 0.05, ns = not significant). (**C**) A heatmap representing taxa SVs that are significantly different between high fructose and control rats. Each column represents different taxa and each row a different sample. Log10 of taxa relative abundance is shown. Results are shown for significant SVs as inferred by a MaAsLin2 analysis (see methods), and the taxonomic classification of each SV is marked at the lowest level available. SVs shown are sorted by the effect size and the number of each SV, as marked in Table S1.

At the taxonomic level, focusing initially on the phylum level, antibiotic intake significantly increased the relative abundance (RA) of Proteobacteria and Bacteroidetes, while reducing the RA of Firmicutes and the overall Firmicutes to Bacteroidetes ratio (Mann Whitney U test, *p* < 5 × 10^−8^ between samples with and without antibiotics, Figure 4A,B). HFrD rats treated with antibiotics had an even higher significant Proteobacteria abundance (Mann Whitney U test, *p* = 7.99 × 10^−3^ between Ctrl + AB and Fructose + AB), while Firmicutes and Bacteroidetes RA showed no significant difference (Mann Whitney U test, *p* > 0.41 between Ctrl + AB and Fructose + AB). Rats given HFrD without antibiotics showed a significant increase in Bacteroidetes RA (Mann Whitney U test, *p* = 3.78 × 10^−2^ between Ctrl and Fructose), and reductions in both Firmicutes RA (Mann Whitney U test, *p* = 6.99 × 10^−3^) and in the Firmicutes to Bacteroidetes ratio (Mann Whitney U test, *p* = 2.62 × 10^−2^), but with no significant difference in Proteobacteria RA (Mann Whitney U test, *p* = 0.80) (Figure 4A,B).

Multivariate association with linear models (MaAsLin2) identified 46 taxa, represented by SV, that were significantly different between the control and fructose samples not treated with antibiotics (see Methods). Most of these taxa SV (37/46) were enriched in fructose samples, and only nine taxa SV had significantly lower RA in fructose compared to control rats (Figure 4C, Table S1). The significantly different taxa were mostly of the Clostridiales order, with 29 of the taxa enriched in fructose and all of the taxa decreased in fructose being of this order. When looking at the differences between control and fructose samples with antibiotics intake, only three taxa SV were significantly different, two of which were from the Proteobacteria phylum and one Bacteroides (Figure S1, Table S2).

### 3.3. SCFA

**Figure 5:**
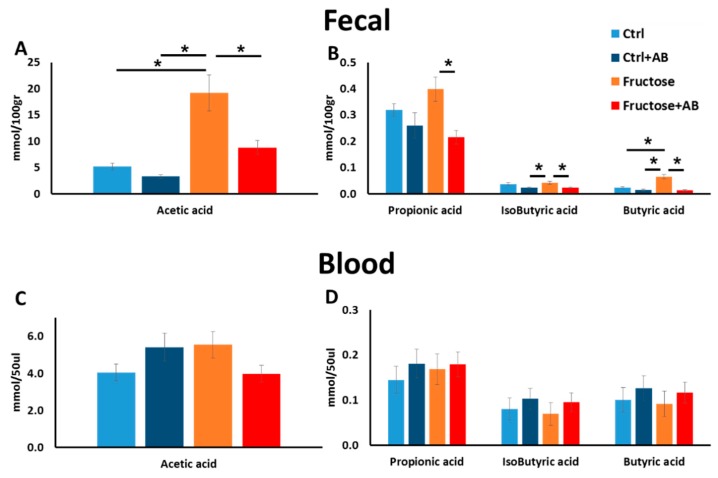
Fructose consumption alters SCFA in the feces but not in blood. SCFA was measured in fecal (**A**,**B**) and blood (**C**,**D**) samples at the end of the experiment.

One mechanism by which the microbiota affects human health and disease is their capacity to produce SCFA by fermenting dietary fibers. Using GC−MS, we measured the blood and feces concentration of the following SCFAs: acetate, propionate, isobutyrate, and butyrate. In the fecal samples, we noted a significant increase in the concentration of acetic and butyric acids in HFrD, while antibiotic treatment of HFrD rats significantly abolished such an effect (Figure 5A,B). The propionic and isobutyric acids concentrations were significantly lower in HFrD rats treated with antibiotics compared to HFrD alone but no difference was seen between HFrD and the control diet (Figure 5B). To capture the systemic effect on host end organ metabolic features, we also measured SCFA concentration in the blood stream. Surprisingly, no differences were seen in the blood samples between all four groups for all four measured SCFAs (Figure 5C,D).

#### 3.3.1. Fatty Liver Phenotype

**Figure 6:**
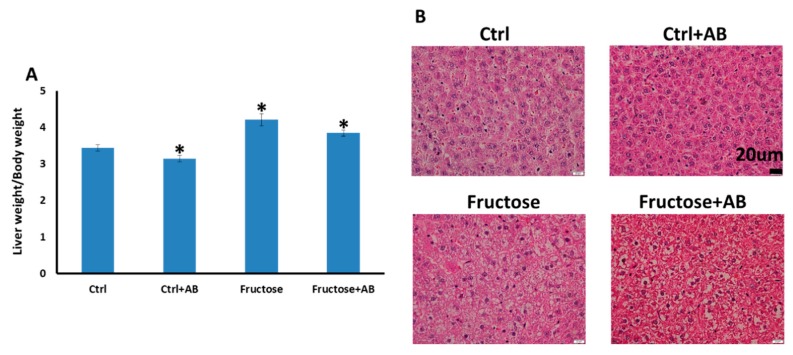
Rats fed HFrD developed fatty liver even with antibiotics treatment. (**A**) Livers were weighed and body weight was determined for standardization *—*p* ≤ 0.05 vs. control. (**B**) Representative slides, hematoxylin eosin staining of liver tissue showing steatosis changes only in rats fed HFrD with or without antibiotics treatment (40× magnification).

HFrD fed rats exhibited heavier livers than the control rats. The HFrD group treated with antibiotics also had significantly heavier livers compared to the controls, but the livers of the control group treated with antibiotics had less mass compared to the controls (Figure 6A). The histological appearance of the HFrD-fed livers showed clear micro-vesicular steatosis changes in three out of the seven rats and these changes were seen in all seven rats in the HFrD and antibiotics group. For the two control groups, no micro-vesicular steatosis was observed (Figure 6B).

#### 3.3.2. Hepatic Gene Expression

**Figure 7:**
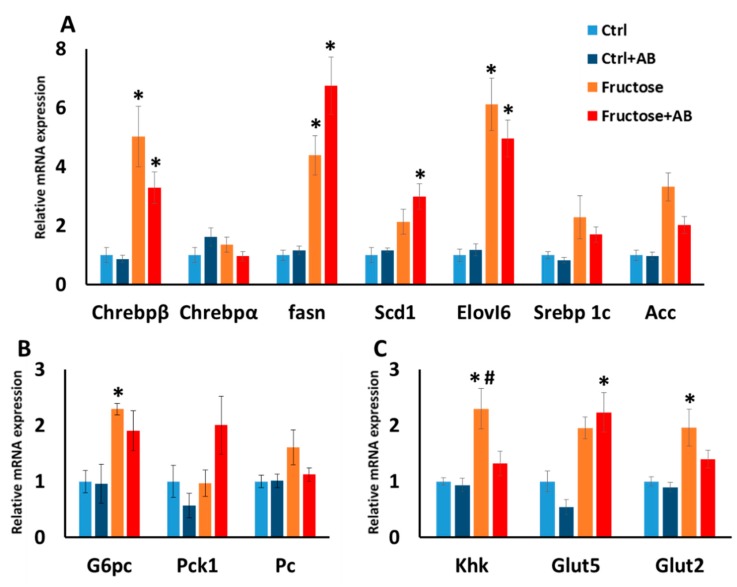
Hepatic gene expression level. Gene expression was measured for lipogenesis genes (**A**), gluconeogenesis genes (**B**) and fructose metabolism and transport genes (**C**). *—*p* ≤ 0.05 versus Ctrl. #—*p* ≤ 0.05 versus Fructose+AB.

Following the fatty liver phenotype, we measured hepatic gene expression of genes related to lipogenesis. HFrD enhanced the expression of Chrebpβ, Fasn and Elovl6 which are key regulator genes in the lipogenesis process, and this enhancement was also seen in HFrD antibiotics treated rats. The upregulation of Scd1 was found in HFrD rats with and without antibiotic treatment, but only in the HFrD antibiotic treated rats did it reach a significant difference (Figure 7A). Additionally, we measured the expression of hepatic genes related to carbohydrate metabolism. For the gluconeogenesis genes, the upregulation of G6pc was found in HFrD rats with and without antibiotic treatment, but only in the HFrD alone rats did it reach a significant difference (Figure 7B). For Khk, the initial enzyme that metabolizes fructose, is was significantly induced in HFrD, and antibiotic treated HFrD rats significantly contrasted this effect. For Glut 5, the main fructose transporter, upregulation was seen in HFrD rats with and without antibiotic treatment but only the HFrD antibiotic treated rats reach a significant difference. For the transporter Glut2, which also transports fructose, HFrD significantly increased its expression and HFrD antibiotic treated rats had levels in between the HFrD and the control rat groups (Figure 7C).

## 4. Discussion

Current Western diets include large amounts of fructose, which have been linked to developing MeS. Fructose consumption also affects the gut microbiome, but it is unclear if and how much the effect on the microbiome mediates the metabolic consequence. For modeling MeS, we fed rats a high fructose diet which induced hypertension, elevated their blood TG and caused fatty liver. We then measured the effect of high fructose on the gut microbiome and on metabolic parameters, and assessed whether antibiotics that substantially reduced microbial diversity and modified the microbial composition prevents the microbial–mediated metabolic effects.

In the control and fructose groups that were not treated with antibiotics, Firmicutes were the most abundant taxa while Bacteroidetes were the second most abundant, and Proteobacteria were hardly represented. This is in agreement with a similar microbial profile reported by Di Luccia et al. [12], using the same animal model. Our high fructose diet versus the control diet showed significantly lower levels of Firmicutes and higher levels of Bacteroidetes, consequently reducing the Firmicutes/Bacteroidetes ratio. These changes in Bacteroidetes and Firmicutes abundance by fructose have also been detected in a recent study on Fischer F344 rats [33]. Additionally, weighted UniFrac-based PCoA plot showed clear clusters that differentiate between the control and fructose fed rats, and multivariate analyses capture of 46 microbiome SVs showed significant differential relative abundances between the two groups. Altogether, this indicates that HFrD alters the microbiome profile. To test the role of the microbial composition on the downstream metabolic effect caused by HFrD, we treated those rats with antibiotics. As expected, antibiotics dramatically reduced microbial diversity, and the relative abundance of Bacteroidetes increased in both HFrD and control rats as was reported by Di Luccia et al. [12]. However, while robust changes were seen in the microbial composition as a result of antibiotic treatment, the effect on the metabolic phenotypes was hardly detected between the HFrD fed groups treated or not with antibiotics.

The most significant physiological phenotypes in the high fructose rat model are fatty liver and the high levels of blood TG. For these two parameters, antibiotics did not reduce the impairments observed in the high fructose diet. High fructose consumption upregulates hepatic genes, which regulate lipogenesis and gluconeogenesis with the most critical transcription factor for those pathways being Chrebpβ [34,35]. In this study, we showed that the upregulation of hepatic Chrebpβ is detected in both high fructose diet and in the antibiotics treated HFrD fed rats, together with Chrebpβ’s downstream genes that participate in lipogenesis and gluconeogenesis. However, antibiotic treatment for the high fructose diet did reduce hepatic Khk level, which is the first enzyme to metabolize fructose, but no accompanied effects were detected in our measurements. Interestingly, in the rats consuming fructose and antibiotics, there were more pronounced histological appearances of clear micro-vesicular steatosis changes than in rats consuming fructose alone. We currently do not have good explanation for this interesting difference. In this study, we observed hypertension in the high fructose group by using the tail cuff measurement and no amelioration was detected after the antibiotic treatment. However, the elevation of insulin and glucose in this model are modest and inconsistent [19,36,37,38] and in our study, HFrD had no effect on those parameters.

We also measured SCFA concentration, which is greatly affected by microbiome activity and can affect the host’s metabolism. In the rats’ fecal matter, high fructose consumption increased acetic and butyric acids concentration (compared to control) while antibiotics treatment for the high fructose group, reduced all four measured SCFA concentration (compared to high fructose without antibiotic treatment). This finding is consistent with the microbiome changes that were detected in the high fructose diet (compared to control) and in the antibiotics treated HFrD rats. In contrast, SCFA concentration in the blood showed no changes between all groups. This finding suggests that the changes in SCFAs induced in high fructose diets and in antibiotics treatment, which both alter the microbiome profile, do not reach the host’s metabolic organs and therefore, probably do not affect the host’s metabolism in a direct manner.

In this study, we observed that although a 60% fructose diet changed the rats′ microbiome profile, antibiotics treatment, which significantly reduced microbial diversity and profile, did not ameliorate the majority of the fructose-associated metabolic impairment. In contrast, previous studies that used a 30% fructose diet in the same rat model and with same antibiotics treatment [1,12,16] have shown a metabolic amelioration. It is possible that the differences in these results lie in the different fructose percentages in the diets. While in the 30% fructose diet, a dramatic intervention in microbiome abundance by antibiotic treatment rescued the metabolic syndrome, in a 60% fructose diet, antibiotic treatment had no effect. This can possibly be due to the different metabolisms of fructose which depend on the fructose dose. In low doses, the small intestine clears most of the dietary fructose, whereas in high doses the intestinal fructose clearance capacity is saturated and the fructose “spills over” to the liver and other organs [39]. Thus, whereas in a 30% fructose diet, the microbiome has a critical role in mediating the metabolic changes, in a 60% fructose diet, the microbiome effect is negligible. Alternatively, it is possible that unlike the 30% fructose diet, the metabolic effect of the 60% fructose diet is due to the induction of gut leakiness that contributes to increased levels of serum bacterial endotoxin lipopolysaccharide (LPS), as was described in previous studies [33]. In this case, the small microbe population, which survived the antibiotics, mediated the metabolic changes and that the specific microbiome profile is less important. To test these two possibilities, further studies using germ free rats consuming 60% fructose are warranted.

## 5. Conclusions

In conclusion, in the rat metabolic syndrome model induced by 60% fructose containing diet, HFrD increased BP and plasma TG levels and induced fatty liver accompanied by changes in the microbiome profile. Antibiotic treatment, which significantly affected the microbial profile, did not change the hemodynamic and metabolic impairments.

## Figures and Tables

**Table 1:** Detailed comparison of the diets.

	**Control (TD.170308) (g/Kg)**		**HFrD (TD.89247) (g/Kg)**	
**Casein**	207		207	
**DL-Methionine**	3		3	
**Corn starch**	400.15		---	
**Maltodextrin**	200		---	
**Fructose**	---		600	
**Lard**	50		50	
**Cellulose**	79.81		79.81	
**Mineral mix rogers-harper (170760)**	50		50	
**Zinc carbonate**	0.04		0.04	
**Vitamin mix Teklad (40,060)**	10		10	
	**% by weight**	**% kcal from**	**% by weight**	**% kcal from**
**Protein**	18.3	21.4	18.3	20.2
**Carbohydrate**	55.4	64.9	60.4	66.8
**fat**	5.2	13.7	5.2	13
**Kcal/g**	3.4		3.6	

**Table 2:** List of primers used in this study.

Gene Name	Forward	Reverse
Chrebpβ	TCTGCAGATCGCGCGGAG	CTTGTCCCGGCATAGCAAC
Chrebpα	TGCATCGATCACAGGTCATT	AGGCTCAAGCATTCGAAGAG
Srebp 1c	AGTTCCAGCATGGCTACCAC	GGGGTCTCTCAGTTTCCTGC
Scd1	TGCTCTGGGGGATATTTTACTACC	GAGAAGAAAAAGCCACGGCG
Elovl6	GAGGCGCAGAGAACACGTAG	CGCTTGTTCATCAGATGCCG
Fasn	AGCCTGAGCTTGTCCCTAGA	CACTGGTACACTTTCCCGCT
Acc	CTTGGGGTGATGCTCCCATT	GCTGGGCTTAAACCCCTCAT
G6pc	CGTCACCTGTGAGACTGGAC	ACGACATTCAAGCACCGGAA
pck1	GGATGTGGCCAGGATCGAAA	ATACATGGTGCGGCCTTTCA
Pc	CCAAGCAGGTTGGCTATGAGAA	GATGTTTTCCTGCCGCAGCC
Khk	ATGGCCATGTTGCCGACTT	TCTGGCAGGTTCGTGTCGTA
Glut5	CATGGTCACGGTTTTTGTGG	AGACGATGCTGACATAGGGC
Glut2	CGCACGCAACATGTCAGAAG	TTATTACCTCTTGAGGTGCATTGA

## Supplementary Materials

The following are available online at https://www.mdpi.com/2072-6643/12/1/203/s1, Figure S1: Specific taxonomic differences between high fructose and control rats, both with antibiotics. Table S1: MaAsLin2 analysis results for comparison between microbial abundance and diet (control against fructose), without antibiotics. Table S2: MaAsLin2 analysis results for comparison between microbial abundance and diet (control against fructose), with antibiotics.

## References

[1] Yerlikaya A., Dagel T., King C., Kubawara M., Lanaspa M.A., Andres-Hernando A., Covic A., Manitius J., Sag A.A., Kanbay M. (2017). Dietary and commercialized fructose: Sweet or sour?. Int. Urol. Nephrol..

[2] Heidenreich P.A., Trogdon J.G., Khavjou O.A., Butler J., Dracup K., Ezekowitz M.D., Finkelstein E.A., Hong Y., Johnston S.C., Khera A. (2011). Forecasting the future of cardiovascular disease in the United States: A policy statement from the American Heart Association. Circulation.

[3] Benjamin E.J., Blaha M.J., Chiuve S.E., Cushman M., Das S.R., Deo R., de Ferranti S.D., Floyd J., Fornage M., Gillespie C. (2017). Heart Disease and Stroke Statistics—2017 Update: A Report From the American Heart Association. Circulation.

[4] Prabhakaran D., Roy A., Praveen P.A., Ramakrishnan L., Gupta R., Amarchand R., Kondal D., Singh K., Sharma M., Shukla D.K. (2017). 20-Year Trend of CVD Risk Factors: Urban and Rural National Capital Region of India. Glob. Heart.

[5] Dekker M.J., Su Q., Baker C., Rutledge A.C., Adeli K. (2010). Fructose: A highly lipogenic nutrient implicated in insulin resistance, hepatic steatosis, and the metabolic syndrome. Am. J. Physiol. Endocrinol. Metab..

[6] Stanhope K.L., Schwarz J.M., Keim N.L., Griffen S.C., Bremer A.A., Graham J.L., Hatcher B., Cox C.L., Dyachenko A., Zhang W. (2009). Consuming fructose-sweetened, not glucose-sweetened, beverages increase visceral adiposity and lipids and decrease insulin sensitivity in overweight/obese men. J. Clin. Investig..

[7] Stanhope K.L., Schwarz J.M., Havel P.J. (2013). Adverse metabolic effects of dietary fructose: Results from the recent epidemiological, clinical, and mechanistic studies. Curr. Opin. Lipidol..

[8] Hannou S.A., Haslam D.E., McKeown N.M., Herman M.A. (2018). Fructose metabolism and metabolic disease. J. Clin. Investig..

[9] Zmora N., Suez J., Elinav E. (2019). You are what you eat: Diet, health and the gut microbiota. Nat. Rev. Gastroenterol. Hepatol..

[10] Do M.H., Lee E., Oh M.J., Kim Y., Park H.Y. (2018). High-Glucose or -Fructose Diet Cause Changes of the Gut Microbiota and Metabolic Disorders in Mice without Body Weight Change. Nutrients.

[11] Mastrocola R., Ferrocino I., Liberto E., Chiazza F., Cento A.S., Collotta D., Querio G., Nigro D., Bitonto V., Cutrin J.C. (2018). Fructose liquid and solid formulations differently affect gut integrity, microbiota composition and related liver toxicity: A comparative in vivo study. J. Nutr. Biochem..

[12] Di Luccia B., Crescenzo R., Mazzoli A., Cigliano L., Venditti P., Walser J.C., Widmer A., Baccigalupi L., Ricca E., Iossa S. (2015). Rescue of fructose-induced metabolic syndrome by antibiotics or faecal transplantation in a rat model of obesity. PLoS ONE.

[13] Sellmann C., Priebs J., Landmann M., Degen C., Engstler A.J., Jin C.J., Garttner S., Spruss A., Huber O., Bergheim I. (2015). Diets rich in fructose, fat or fructose and fat alter intestinal barrier function and lead to the development of nonalcoholic fatty liver disease over time. J. Nutr. Biochem..

[14] Volynets V., Louis S., Pretz D., Lang L., Ostaff M.J., Wehkamp J., Bischoff S.C. (2017). Intestinal Barrier Function and the Gut Microbiome Are Differentially Affected in Mice Fed a Western-Style Diet or Drinking Water Supplemented with Fructose. J. Nutr..

[15] Bergheim I., Weber S., Vos M., Kramer S., Volynets V., Kaserouni S., McClain C.J., Bischoff S.C. (2008). Antibiotics protect against fructose-induced hepatic lipid accumulation in mice: role of endotoxin. J. Hepatol..

[16] Crescenzo R., Mazzoli A., Di Luccia B., Bianco F., Cancelliere R., Cigliano L., Liverini G., Baccigalupi L., Iossa S. (2017). Dietary fructose causes defective insulin signalling and ceramide accumulation in the liver that can be reversed by gut microbiota modulation. Food Nutr. Res..

[17] Li J.M., Yu R., Zhang L.P., Wen S.Y., Wang S.J., Zhang X.Y., Xu Q., Kong L.D. (2019). Dietary fructose-induced gut dysbiosis promotes mouse hippocampal neuroinflammation: A benefit of short-chain fatty acids. Microbiome.

[18] Sanchez-Lozada L.G., Tapia E., Jimenez A., Bautista P., Cristobal M., Nepomuceno T., Soto V., Avila-Casado C., Nakagawa T., Johnson R.J. (2007). Fructose-induced metabolic syndrome is associated with glomerular hypertension and renal microvascular damage in rats. Am. J. Physiol. Renal. Physiol..

[19] Wong S.K., Chin K.Y., Suhaimi F.H., Fairus A., Ima-Nirwana S. (2016). Animal models of metabolic syndrome: A review. Nutr. Metab..

[20] Kucera O., Cervinkova Z. (2014). Experimental models of non-alcoholic fatty liver disease in rats. World J. Gastroenterol..

[21] Kawasaki T., Igarashi K., Koeda T., Sugimoto K., Nakagawa K., Hayashi S., Yamaji R., Inui H., Fukusato T., Yamanouchi T. (2009). Rats fed fructose-enriched diets have characteristics of nonalcoholic hepatic steatosis. J. Nutr..

[22] Ferrier L., Berard F., Debrauwer L., Chabo C., Langella P., Bueno L., Fioramonti J. (2006). Impairment of the intestinal barrier by ethanol involves enteric microflora and mast cell activation in rodents. Am. J. Pathol..

[23] Kamari Y., Shaish A., Vax E., Shemesh S., Kandel-Kfir M., Arbel Y., Olteanu S., Barshack I., Dotan S., Voronov E. (2011). Lack of interleukin-1alpha or interleukin-1beta inhibits transformation of steatosis to steatohepatitis and liver fibrosis in hypercholesterolemic mice. J. Hepatol..

[24] Braun T., Di Segni A., BenShoshan M., Asaf R., Squires J.E., Farage Barhom S., Glick Saar E., Cesarkas K., Smollan G., Weiss B. (2017). Fecal microbial characterization of hospitalized patients with suspected infectious diarrhea shows significant dysbiosis. Sci. Rep..

[25] Caporaso J.G., Lauber C.L., Walters W.A., Berg-Lyons D., Huntley J., Fierer N., Owens S.M., Betley J., Fraser L., Bauer M. (2012). Ultra-high-throughput microbial community analysis on the Illumina HiSeq and MiSeq platforms. ISME J..

[26] Flores G.E., Henley J.B., Fierer N. (2012). A direct PCR approach to accelerate analyses of human-associated microbial communities. PLoS ONE.

[27] Caporaso J.G., Kuczynski J., Stombaugh J., Bittinger K., Bushman F.D., Costello E.K., Fierer N., Pena A.G., Goodrich J.K., Gordon J.I. (2010). QIIME allows analysis of high-throughput community sequencing data. Nat. Methods.

[28] Amir A., McDonald D., Navas-Molina J.A., Kopylova E., Morton J.T., Zech Xu Z., Kightley E.P., Thompson L.R., Hyde E.R., Gonzalez A. (2017). Deblur Rapidly Resolves Single-Nucleotide Community Sequence Patterns. mSystems.

[29] Lozupone C., Knight R. (2005). UniFrac: A new phylogenetic method for comparing microbial communities. Appl. Environ. Microbiol..

[30] Mirarab S., Nguyen N., Warnow T. (2012). SEPP: SATe-enabled phylogenetic placement. Pac. Symp. Biocomput..

[31] Xu Z.Z., Amir A., Sanders J., Zhu Q., Morton J.T., Bletz M.C., Tripathi A., Huang S., McDonald D., Jiang L. (2019). Calour: An Interactive, Microbe-Centric Analysis Tool. mSystems.

[32] Morgan X.C., Tickle T.L., Sokol H., Gevers D., Devaney K.L., Ward D.V., Reyes J.A., Shah S.A., LeLeiko N., Snapper S.B. (2012). Dysfunction of the intestinal microbiome in inflammatory bowel disease and treatment. Genome Biol..

[33] Cho Y.E., Kim D.K., Seo W., Gao B., Yoo S.H., Song B.J. (2019). Fructose Promotes Leaky Gut, Endotoxemia, and Liver Fibrosis Through Ethanol-Inducible Cytochrome P450-2E1-Mediated Oxidative and Nitrative Stress. Hepatology.

[34] Kim M.S., Krawczyk S.A., Doridot L., Fowler A.J., Wang J.X., Trauger S.A., Noh H.L., Kang H.J., Meissen J.K., Blatnik M. (2016). ChREBP regulates fructose-induced glucose production independently of insulin signaling. J. Clin. Investig..

[35] Kim M., Astapova I.I., Flier S.N., Hannou S.A., Doridot L., Sargsyan A., Kou H.H., Fowler A.J., Liang G., Herman M.A. (2017). Intestinal, but not hepatic, ChREBP is required for fructose tolerance. JCI Insight.

[36] Burgeiro A., Cerqueira M.G., Varela-Rodriguez B.M., Nunes S., Neto P., Pereira F.C., Reis F., Carvalho E. (2017). Glucose and Lipid Dysmetabolism in a Rat Model of Prediabetes Induced by a High-Sucrose Diet. Nutrients.

[37] Lozano I., Van der Werf R., Bietiger W., Seyfritz E., Peronet C., Pinget M., Jeandidier N., Maillard E., Marchioni E., Sigrist S. (2016). High-fructose and high-fat diet-induced disorders in rats: Impact on diabetes risk, hepatic and vascular complications. Nutr. Metab..

[38] D’Angelo G., Elmarakby A.A., Pollock D.M., Stepp D.W. (2005). Fructose feeding increases insulin resistance but not blood pressure in Sprague-Dawley rats. Hypertens (Dallas, Tex 1979).

[39] Jang C., Hui S., Lu W., Cowan A.J., Morscher R.J., Lee G., Liu W., Tesz G.J., Birnbaum M.J., Rabinowitz J.D. (2018). The Small Intestine Converts Dietary Fructose into Glucose and Organic Acids. Cell Metab..

