# Supplementary figures and images for: Antibiotic Treatment Does Not Ameliorate the Metabolic Changes in Rats Presenting Dysbiosis After Consuming a High Fructose Diet
Source: Nutrients. 2020 Jan 13;12(1):203. doi: 10.3390/nu12010203 (PMC7019627; doi:10.3390/nu12010203)

## Slide 1
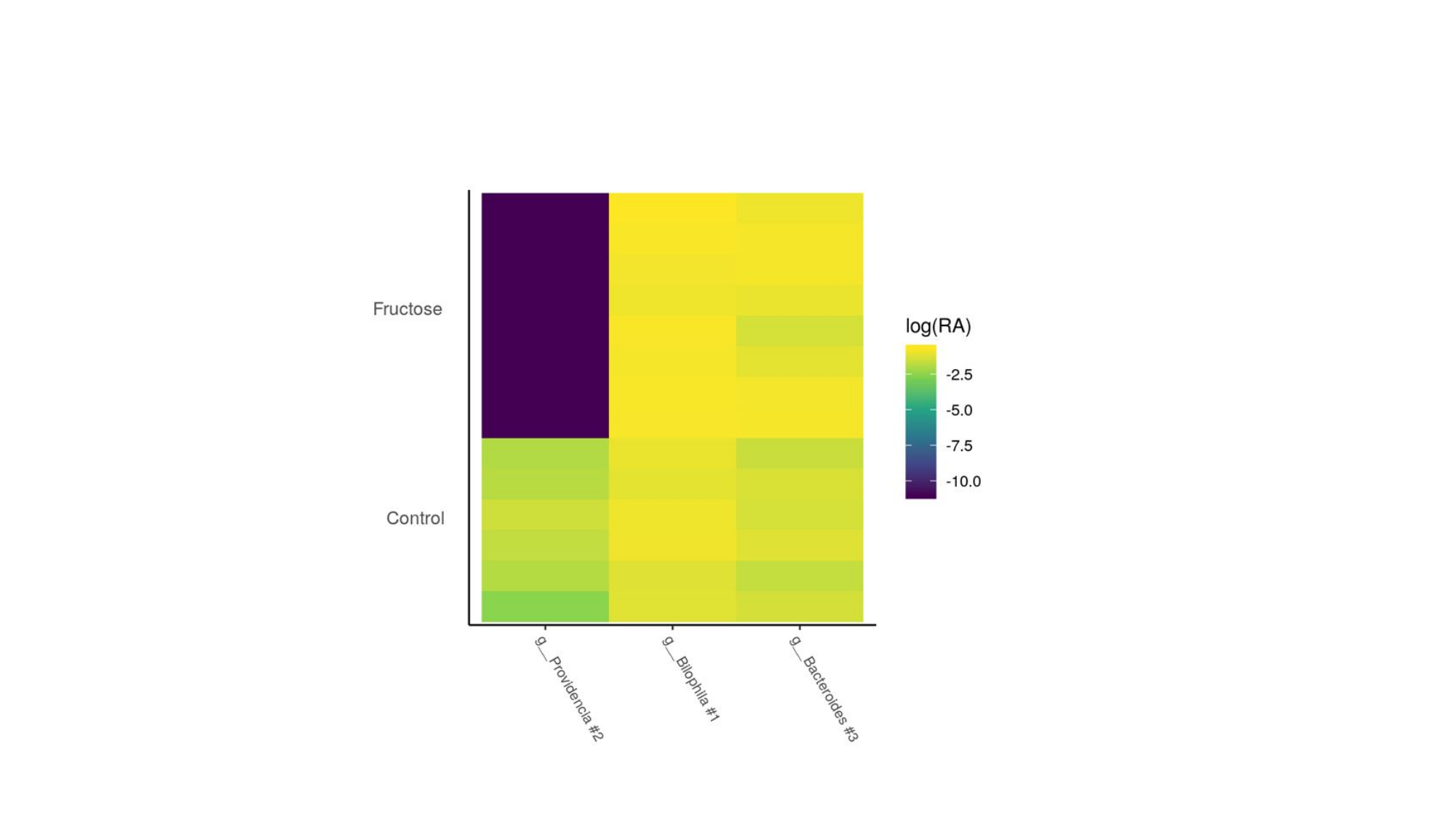

Supplement: Supplementary file 1 [file nutrients-12-00203-s001.zip › SUPP/Supplementary figure 1.pptx]
